# Centromere drive may propel the evolution of chromosome and genome size in plants

**DOI:** 10.1093/aob/mcae149

**Published:** 2024-08-28

**Authors:** Klára Plačková, Petr Bureš, Martin A Lysak, František Zedek

**Affiliations:** Department of Botany and Zoology, Faculty of Science, Masaryk University, Kotlarska 2, 611 37 Brno, Czech Republic; Department of Botany and Zoology, Faculty of Science, Masaryk University, Kotlarska 2, 611 37 Brno, Czech Republic; CEITEC – Central European Institute of Technology, Masaryk University, Kamenice 5, 625 00 Brno, Czech Republic; Department of Botany and Zoology, Faculty of Science, Masaryk University, Kotlarska 2, 611 37 Brno, Czech Republic

**Keywords:** Angiosperms, asymmetric and symmetric meiosis, bryophytes, CENH3, centromere drive, chromosome size, ferns, genome size, gymnosperms, lycophytes, post-polyploid diploidization

## Abstract

**Background:**

Genome size is influenced by natural selection and genetic drift acting on variations from polyploidy and repetitive DNA sequences. We hypothesized that centromere drive, where centromeres compete for inclusion in the functional gamete during meiosis, may also affect genome and chromosome size. This competition occurs in asymmetric meiosis, where only one of the four meiotic products becomes a gamete. If centromere drive influences chromosome size evolution, it may also impact post-polyploid diploidization, where a polyploid genome is restructured to function more like a diploid through chromosomal rearrangements, including fusions. We tested if plant lineages with asymmetric meiosis exhibit faster chromosome size evolution compared to those with only symmetric meiosis, which lack centromere drive as all four meiotic products become gametes. We also examined if positive selection on centromeric histone H3 (CENH3), a protein that can suppress centromere drive, is more frequent in these asymmetric lineages.

**Methods:**

We analysed plant groups with different meiotic modes: asymmetric in gymnosperms and angiosperms, and symmetric in bryophytes, lycophytes and ferns. We selected species based on available CENH3 gene sequences and chromosome size data. Using Ornstein–Uhlenbeck evolutionary models and phylogenetic regressions, we assessed the rates of chromosome size evolution and the frequency of positive selection on CENH3 in these clades.

**Results:**

Our analyses showed that clades with asymmetric meiosis have a higher frequency of positive selection on CENH3 and increased rates of chromosome size evolution compared to symmetric clades.

**Conclusions:**

Our findings support the hypothesis that centromere drive accelerates chromosome and genome size evolution, potentially also influencing the process of post-polyploid diploidization. We propose a model which in a single framework helps explain the stability of chromosome size in symmetric lineages (bryophytes, lycophytes and ferns) and its variability in asymmetric lineages (gymnosperms and angiosperms), providing a foundation for future research in plant genome evolution.

## INTRODUCTION

Genome size, the amount of DNA in the cell nucleus, is a relatively stable species trait (Šmarda and Bureš, 2010; Pellicer *et al.*, 2018) that is affected by environmental and demographic factors (Bureš *et al.*, 2024; Soto Gomez *et al.*, 2024), which ultimately determine whether its evolution is driven by natural selection or genetic drift (Lynch and Conery, 2003; Knight *et al.*, 2005; Faizullah *et al.*, 2021). Depending on the context, natural selection or genetic drift can lead to the fixation of genome size changes caused primarily by whole genome duplications (polyploidy) or the activity of transposable elements (TEs), satellite DNA and other repeats, the main proximal mechanisms generating genome size variation (Kubis *et al.*, 1998; Leitch and Leitch, 2008; Tenaillon *et al.*, 2010; Lisch, 2013; Bennetzen and Wang, 2014; Šmarda *et al.*, 2019).

Another deterministic evolutionary mechanism besides natural selection is meiotic drive, whereby selfish genetic elements alter chromosome segregation during meiosis (Sandler and Novitski, 1957), so that they are passed on to the next generation more frequently than expected by Mendel’s rule (Clark and Akera, 2021). This often occurs in asymmetric meiosis, where only one of the resulting meiotic products becomes a gamete, while the other three degenerate and do not participate in reproduction (Lenormand *et al.*, 2016). In contrast, symmetric meiosis results in all four meiotic products becoming gametes (Lenormand *et al.*, 2016). Asymmetric meiosis thus provides an arena for competition among genetic elements for inclusion in the sole surviving meiotic product. If the success of such a genetic element depends on its size, meiotic drive could influence the evolution of genome size (Talbert *et al.*, 2009; Bureš and Zedek, 2014; Dawe, 2022; Plačková *et al.*, 2022).

For example, centromere drive, in which homologous centromeres in asymmetric meiosis compete with each other based on their size, propels the evolution of centromere size (Henikoff *et al.*, 2001; Rosin and Mellone, 2017; Dudka and Lampson, 2022; Talbert and Henikoff, 2022). It appears that the larger of the homologous centromeres is predominantly captured by the pole that has more microtubule-organizing centres and a higher microtubule density (Dudka and Lampson, 2022; Talbert and Henikoff, 2022). If this pole corresponds to the egg pole, the larger centromere is more frequently positioned within the egg (Chmatal *et al.*, 2014, 2017; Iwata-Otsubo *et al.*, 2017) and thus preferred by centromere drive. In contrast, if it is the polar body pole, the larger centromere tends to reside within the polar body, while the smaller centromere is more likely to be positioned in the egg (Wu *et al.*, 2018). In this scenario, the smaller centromere would therefore be favoured by centromere drive. Hereafter, we use the expressions ‘centromere drive prefers’ and ‘centromere drive favours’ larger or smaller centromeres to simplify the scenarios explained above, without implying any form of agency.

By rapidly fixing changes in the size of the centromere (Finseth *et al.*, 2021), whose expansion and contraction can occur relatively easily (Showman *et al.*, 2024), centromere drive obviously affects chromosome and genome sizes themselves. However, the effects of centromere drive on chromosome and genome size may be more complex, as it appears that the size of the active centromere may determine the range of chromosome sizes (Plačková *et al.*, 2022). A positive relationship between the total centromere/kinetochore size and genome size has been repeatedly demonstrated in grasses (Bennett *et al.*, 1981; Zhang and Dawe, 2012; Wang *et al.*, 2021) and later confirmed across eukaryotes (Plačková *et al.*, 2021). Although less attention has been paid to the intrakaryotype relationship between the sizes of individual centromeres/kinetochores and their respective chromosomes, the positive relationship at this level has been documented in humans (Irvine *et al.*, 2004), maize (Wang *et al.*, 2021) and species of the plant subfamily Agavoideae (Plačková *et al.*, 2022). The larger centromeres/kinetochores allow the attachment of more microtubules and the chromosome can thus be pulled by a greater force (reviewed in Plačková *et al.*, 2022), which helps the larger chromosomes to overcome the stronger frictional resistance of non-kinetochore microtubules (Houchmandzadeh *et al.*, 1997; Shimamoto *et al.*, 2011; Kramer *et al.*, 2021) and highly viscous cytoplasm (Nicklas, 1965; Ui *et al.*, 1984). Based on these findings, we hypothesized that the centromere’s size limits the size of chromosomes and that centromere drive could propel the evolution of chromosome/genome size by rapidly driving changes in centromere size to fixation (Plačková *et al.*, 2022). Moreover, centromere drive can also influence the process of post-polyploid diploidization (PPD) through its effects on centromere and chromosome size. PPD involves multi-level genomic changes that act on a polyploid genome and gradually restore a diploid-like state and disomic inheritance. Diploidization, typically mediated by intra- and interchromosomal recombination, leads to gene fractionation, gene and repeat losses, and significant changes in chromosome structure, size and number including the origin of fusion chromosomes (Mandáková and Lysak, 2018; Li *et al.*, 2021).

Here we aim to address the hypothesis of centromere drive’s role in chromosome and genome size evolution by testing whether plant lineages with centromere drive capability exhibit a more rapid evolution of chromosome size than lineages without centromere drive. We define the opportunity for centromere drive by the presence of asymmetric meiosis, in which only one meiotic product becomes a gamete while the remaining three degenerate. In this sense, angiosperms and gymnosperms have asymmetric female meiosis ( Williams 2009; Boavida and McCormick, 2010; Gorelick *et al.*, 2017), and thus the opportunity for centromere drive (Henikoff *et al.*, 2001; Talbert and Henikoff, 2022). On the other hand, cryptogamous plants such as ferns, lycophytes or bryophytes have symmetric meiosis regardless of the sex (Brown and Lemmon, 2013; Lenormand *et al.*, 2016; Gorelick *et al.*, 2017), meaning that all four meiotic products survive and become gametes, and therefore there is no opportunity for centromere drive (Henikoff *et al.*, 2001; Kinosian *et al.*, 2022; Talbert and Henikoff, 2022). Although bryophytes do not differentiate between male and female meiosis due to their non-specialized sporophyte generation (Gorelick *et al.*, 2017), all of their four meiotic products survive (Brown and Lemmon, 2013), aligning them with other symmetrics because there is nothing to compete for. In addition to assessing the rate of chromosome size evolution, we also examine the frequency of positive selection acting on centromeric histone H3 (CENH3), a key protein involved in kinetochore function during homologous chromosome segregation in mitosis and meiosis (Talbert and Henikoff, 2022). CENH3 mutations, which alter its loading to centromeres and restore meiotic parity between homologous centromeres, suppress centromere drive and are positively selected (Zedek and Bureš, 2016*a*; Finseth *et al.*, 2021; Talbert and Henikoff, 2022). Consistent with the centromere drive model, previous research has shown that CENH3 is more frequently under positive selection in eukaryotes with asymmetric meiosis (Zedek and Bureš, 2016*a*). By focusing on plants and using larger species datasets, we reassess whether the predicted differential evolution of CENH3 is manifested in clades belonging to angiosperms and gymnosperms (all having asymmetric female meiosis; henceforth referred to as asymmetric clades) versus clades belonging to bryophytes, lycophytes and ferns (all having only symmetric meiosis; henceforth referred to as symmetric clades).

## METHODS

### General methodical approach and analysed species and clades

We examined plant species with asymmetric female meiosis (gymnosperms and angiosperms) and only symmetric meiosis (bryophytes, lycophytes and ferns). To select the species for our analysis, we only considered those for which the CENH3 gene sequence was available from previous studies (Zedek and Bureš, 2016*a*, *b*; Krátká *et al.*, 2021) or could be obtained by BLAST searches in the public databases GenBank (Benson *et al.*, 2005) and China National GeneBank (Chen *et al.*, 2020). We grouped these species into clades at different taxonomic levels (genus, family, order or class; Supplementary Data Table S1). Then we collected data on genome size and chromosome number – mainly from Bureš *et al*. (2024), Pellicer and Leitch (2020) and Rice *et al.* (2015) – for as many species of the selected clades as possible. The mean chromosome size for species calculated from genome size and chromosome count enabled us to assess the rates of chromosome size evolution in the analysed clades. We obtained a total of 384 species from 27 clades based on these criteria for further analyses. The list of analysed species and clades, including all data sources, can be found in Tables S1 and S2.

### Inference of the frequency of positive selection on CENH3 in each clade

To estimate the frequency of positive selection on CENH3 in each clade, we used a method that involved identifying positively selected branches and codons in the CENH3 gene phylogeny and coding sequence, respectively. First, we obtained CENH3 codon alignments (Supplementary Data Table S3) for each clade using the MAFFT algorithm implemented on the MAFFT Server (Katoh *et al.*, 2019). To identify potential paralogues of CENH3, which could have originated either through polyploidy or gene duplication events within clades, we utilized the ProteinOrtho program (Lechner *et al*., 2011) to analyse each alignment. Additionally, we supplemented this analysis by visually comparing the CENH3 gene tree with the clade species tree (refer to Table S4). The information obtained regarding the numbers of paralogous and orthologous branches in CENH3 gene trees (File S1) was later considered in the calculation of the frequency of positive selection acting on CENH3 (see below).

Then, we used two codon substitution models on the Datamonkey 2.0 webserver (Weaver *et al.*, 2018) to determine selective pressures acting on the full-length CENH3, and N-terminal and C-terminal parts of CENH3 (Supplementary Data Tables S5 and S6). To identify positively selected branches, we employed the adaptive Branch-Site Random Effects Likelihood model (aBSREL; Smith *et al.*, 2015), which independently evaluates selective pressures acting on individual branches in a given phylogeny (Table S4) and provides a branch-level non-synonymous/synonymous substitution rate ratio (dN/dS = ω; Table S2). To identify positively selected codons, we employed the Mixed Effects Model of Evolution (MEME; Murrell *et al.*, 2012), which independently infers selective regimes at the level of individual codons and provides a codon-level dN/dS ratio (Table S2). To calculate the frequency of positively selected branches, we divided the number of positively selected branches (with an uncorrected *P*-value <0.05) by the number of branches of orthologous groups (Table S2), thereby taking into account the independent evolution of paralogues. To calculate the frequency of positively selected codons, we divided the number of positively selected codons (with a *P*-value threshold of 0.1) by the number of codons of aligned sequences. The uncorrected *P*-values from aBSREL and the *P*-value threshold of 0.1 in MEME were chosen based on recommendations for exploratory analyses (Spielman *et al.*, 2019). Finally, we multiplied the frequency of positively selected branches and codons to obtain a single number for each clade (Table S2). To test the difference between dN/dS ratios, we used the Mann–Whitney test.

### Inference of the rate of chromosome size evolution in each clade

To evaluate the rate of chromosome size evolution in each clade, we started by collecting genome sizes (2C in Mb) and chromosome numbers (2*n*) for each species mainly from Bureš *et al.* (2024), Plant C-values Database (Pellicer and Leitch, 2020) and Chromosome Count Database (Rice *et al.*, 2015). Next, we calculated the mean chromosome size for each species as the ratio of 2C to 2*n*. To ensure that changes in mean chromosome size were comparable across clades, we standardized the mean chromosome sizes within each clade by z-transformation using the ‘scale’ function in base R v.4.2.2 (R Core Team, 2022). To assess the rate of chromosome size evolution within each clade, we utilized phylogenetic independent contrasts (PICs) on the z-transformed mean chromosome sizes. We applied the ‘pic’ function in the R package ape (Paradis and Schliep, 2019) to obtain PICs, and then calculated the median of the absolute values of the PICs to obtain a single value of the rate of chromosome size evolution for each clade (Supplementary Data Table S2). We schematically summarized this procedure on examples from Asteraceae and Pteridaceae in Fig. S1. To obtain ultrametric clade-specific phylogenetic trees for the PIC analysis (Table S4), we pruned phylogenies from TimeTree (Kumar *et al.*, 2022) or species-level phylogenetic trees for all angiosperm species (Bureš *et al.*, 2024) to contain only the species in our dataset. The species-level trees for each clade as well as the clade-level phylogenetic tree are provided in Table S4. The clade-level phylogenetic tree was visualized using the R package ggtree (Yu *et al*., 2017).

### Assessing differences in the frequency of positive selection on CENH3 and chromosome size evolution between asymmetrics and symmetrics

We employed two approaches to investigate differences in the frequency of positive selection acting on CENH3 and the rate of chromosome size evolution between asymmetric and symmetric clades. First, a regression analysis utilizing phylogenetic generalized linear models (PGLS) was conducted in the R package caper v.1.0.3 (Orme *et al*., 2023). We set the frequency of positive selection acting on CENH3 and the rate of chromosome evolution as response variables, and the presence/absence of centromere drive as a predictor. We applied the square-root transformation on the response variables to ensure the homoscedasticity of the residuals. In the second approach, we employed Ornstein–Uhlenbeck models (Hansen, 1997; Butler and King, 2004) implemented in the R package OUwie v.2.10 (Beaulieu and OʹMeara, 2022). OUwie is a statistical package that allows testing for the existence of an optimum towards which a trait is being pulled. The optimum is represented by a central point, which is referred to as theta (θ). The strength with which the trait is being pulled towards θ is quantified by the alpha parameter (α), while the random fluctuations around θ are captured by sigma squared (σ^2^). In our study, we aimed to estimate θ to determine the optimal value towards which the rate of chromosome size evolution and frequency of positive selection on CENH3 is evolving in symmetric and asymmetric clades. We considered seven different models, including two Brownian motion models (BM1 and BMS), which assume constant variance of the trait through time, and five Ornstein–Uhlenbeck models (OU1, OUM, OUMV, OUMA and OUMVA). While BM1 is a basic Brownian motion model, BMS is an extended model with rate parameters for stabilizing or directional selection. OU1 assumes that the trait evolves towards a single θ with constant α and σ^2^ parameters, and OUM allows θ to vary between categories, namely asymmetrics and symmetrics. OUMV allows for variation in both θ and σ^2^, OUMA allows for variation in θ and α, and OUMVA allows for variation in all three parameters. We selected the best models based on the weighted Akaike information criterion (AICw; Akaike, 1978; Wagenmakers and Farrel, 2004) and evidence ratios (i.e. AICw of the first model vs. AICw of other models; Burnham and Anderson, 2002). Instead of choosing the best-fitting model solely based on its absolute AICw value, we applied a rule-of-thumb threshold of 3.0 for the evidence ratio, providing a relative scaling of alternative models. This criterion denotes that a model is considered well-supported relative to another model (Burnham and Anderson, 2002). To assess the significance of the potential difference in θ between asymmetrics and symmetrics, we generated 100 parametric bootstrap replications using the ‘OUwie.boot’ function and tested the difference between the two θ distributions using the Mann–Whitney test. Bootstrapped values of the frequency of positive selection acting on CENH3 and the rate of chromosome size evolution were visualized as density plots using the R package ggplot2 (Wickham, 2016).

Because OU processes are often interpreted in the context of natural selection (Hansen, 1997; Butler and King, 2004), one might argue whether the frequency or rate can indeed be subject to selection. However, it is important to recognize that selection is only one of the biological and other interpretations of the OU process (Blackwell, 2005; Cooper *et al*., 2015). Originally, the OU process was introduced to describe the random motion of a particle influenced by a restoring force towards a central point, just like random forces in physics (Uhlenbeck and Ornstein, 1930). Here we investigated whether the particles (the rate and frequency) are pulled towards different central points (optima in θ) in clades with and without centromere drive. The pulling force is the presence/absence of centromere drive and its impact on the chromosome and CEHN3 evolution (see Introduction).

## RESULTS

**Fig. 1.**
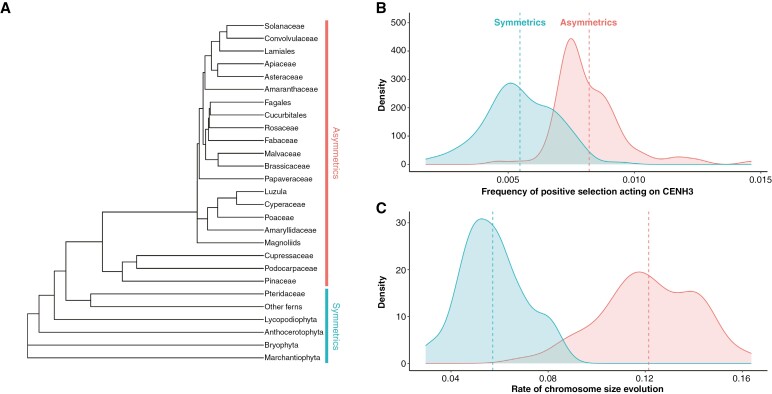
(A) Clade-level phylogenetic tree. (B) Bootstrapped values of the frequency of positive selection acting on CENH3 in clades with female asymmetric meiosis (θ_A_ = 0.008) and symmetric meiosis regardless of the sex (θ_S_ = 0.004) under the OUMA model. (C) Bootstrapped values of chromosome size evolution rate in asymmetrics (θ_A_ = 0.118) and symmetrics (θ_S_ = 0.058) under the OUMA model. Density plots show the distributions of 100 parametric bootstrap replications. The vertical dashed lines in (B) and (C) represent the mean values.

We analysed the frequency of positive selection acting on CENH3 using, in total, 475 sequences from 384 taxa (Supplementary Data Table S1) from 21 clades with asymmetric female meiosis and six clades having only symmetric meiosis (Fig. 1A). From these, 125 sequences (~26 %) were analysed in earlier studies (Zedek and Bureš, 2016*a*, *b*; Krátká *et al*., 2021), while 350 sequences (~74 %) were analysed here for the first time.

Generally, the average dN/dS ratio was higher in asymmetric clades compared to symmetric clades, whether we analysed the full-length CENH3 or the C- and N-terminal parts separately (Supplementary Data Fig. S2; Table S2). This indicated a higher rate of CENH3 evolution in asymmetric clades, which was corroborated by subsequent phylogenetic analyses of the frequency of positive selection acting on CENH3 employing Ornstein–Uhlenbeck models and PGLS.

The best-fitting model in the OUwie analyses was OUMA (Table 1) which showed that the frequency of selection acting on the full-length CENH3 in asymmetrics (θ = 0.007) was significantly higher (*P* < 0.001; Mann–Whitney test) than in symmetrics (θ = 0.003; Fig. 1B; Supplementary Data Table S7). When examining the frequencies of positive selection separately for the C- and N-terminal parts of CENH3, we found differences between the two domains. For the C-terminus, the best-fitting model was OUMV with two different optima, showing a significantly higher (*P* < 0.001; Mann–Whitney test) frequency of selection in asymmetrics (θ = 0.0029) than in symmetrics (θ = 0.0001; Fig. S3a; Table S7). For the N-terminus, the best-fitting model was OU1 with a single optimum of the frequency of selection in both asymmetrics and symmetrics (θ = 0.163; Table S7). After assessing AICw, we identified models for the frequency of selection on the full-length CENH3 (OUMV) and the C-terminal part of CENH3 (OUMA) that exhibited a probability of being comparable to the first best-fit model (evidence rates lower than 3.0; see Methods; Table 1). Both models indicated a higher (*P* < 0.001; Mann–Whitney test) frequency of selection on the whole CENH3 and the C-terminal part of CENH3 in asymmetrics compared to symmetrics (Table S7; Fig. S3b, c). The phylogenetic regressions showed that the frequency of positive selection on the C-terminal part of the CENH3 gene was higher in asymmetrics than in symmetrics (*P* = 0.031; Table S8). However, the difference between asymmetrics and symmetrics was only marginally significant for the full-length CENH3 (*P* = 0.074) and insignificant for the N-terminus (*P* = 0.550).

**Table 1.** Performance of Brownian motion and Ornstein–Uhlenbeck models based on weighted AIC.

	OU1	BM1	BMS	OUM	OUMV	OUMA	OUMVA
Frequency of positive selection acting on full-length CENH3	0.0794	0.0002	0.1195	0.0455	**0.3242**	**0.3603**	0.0709
Frequency of positive selection acting on C-terminus	0.0000	0.0000	0.0007	0.0000	**0.4568**	**0.4566**	0.0858
Frequency of positive selection acting on N-terminus	**0.5840**	0.0869	0.0576	0.1753	0.0479	0.0392	0.0091
Rate of chromosome size evolution	0.0622	0.0002	0.0599	0.0851	**0.3690**	**0.3552**	0.0684

The models with the highest AICw are highlighted in bold. In the case of two bold values, they are considered equally probable based on the evidence rates.

We obtained mean chromosome sizes for 290 of 384 taxa for which we had CENH3 sequences (Supplementary Data Table S1). The best-fitting model for the rates of chromosome size evolution was OUMV (Table 1), showing that asymmetrics have significantly higher (*P* < 0.001; Mann–Whitney test) rates of chromosome size evolution (θ = 0.121) than symmetrics (θ = 0.058; Fig. 1C; Table S7). The second best-fitting model was OUMA (Table 1), where the theta parameters acquired the same values as for the OUMV model (Table S7; Fig. S3d). The phylogenetic regression showed that the rate of chromosome size evolution is increased in asymmetrics compared to symmetrics (*P* = 0.029; Table S8).

## DISCUSSION

### Symmetrics and asymmetrics differ in CENH3, chromosome size and karyotype evolution

We observed that plant clades with asymmetric female meiosis had a higher frequency of positive selection on both the full-length and C-terminal tail of CENH3 than clades with only symmetric meiosis (see Fig. 1B; Supplementary Data Tables S7 and S8). Interestingly, there was no difference in this regard for the N-terminus (Table 1; Tables S7 and S8), despite its greater heterogeneity in length and sequence (Malik and Henikoff, 2003; Maheshwari *et al.*, 2015; Elisafenko *et al.*, 2021). These findings are consistent with the centromere drive model, as the C-terminal region is responsible for loading CENH3 to the centromere (Dalal *et al.*, 2007). Our results are also consistent with previous findings (Zedek and Bureš, 2016*a*, *b*; Krátká *et al*., 2021) and with predictions of the centromere drive model (Henikoff *et al.*, 2001; Talbert and Henikoff, 2022). It is important to note that seed plants (asymmetrics) feature organisms with a dominant diploid generation, whereas non-seed plants (symmetrics) have a life cycle with a dominant haploid generation. This distinction may have influenced our selection analyses, as deleterious mutations in a haploid genome are expected to be more effectively purged due to the absence of masking, while beneficial alleles are more likely to achieve fixation in the population (Otto and Goldstein, 2021). However, contrary to expectations, no systematic difference in the dN/dS ratio has been found between seed and non-seed plants (Stenøien, 2008; Szövényi *et al.*, 2013; Linde *et al.*, 2021).

In addition, we found that asymmetrics have a higher rate of chromosome size evolution than symmetrics (Fig. 1C), corroborating the previously hypothesized effects of centromere drive on chromosome and genome size evolution (Plačková *et al.*, 2022). If centromere drive can indeed accelerate chromosome and genome size evolution through its effects on centromere size, this could help explain the remarkable differences in genome and karyotype evolution between plant asymmetrics (angiosperms, gymnosperms) and symmetrics (bryophytes, ferns, lycophytes; Clark *et al.*, 2016; Zedek and Bureš, 2016*a*; Fujiwara *et al.*, 2023; Bureš *et al.*, 2024). While a clear polyploidy-related positive correlation between genome size and chromosome number can be observed in symmetrics in both closely related species and distantly related species and clades (Nakazato *et al.*, 2008; Haufler, 2014; Clark *et al.*, 2016; Liu *et al.*, 2019), this correlation in asymmetrics is only positive among closely related taxa but becomes slightly negative when considering a broader phylogenetic scale (Choi *et al.*, 2020; Roddy *et al.*, 2020). This inversion of the correlation between genome size and chromosome number in asymmetrics is probably due to chromosome fusions during post-polyploid diploidization (Mandáková and Lysak, 2018; Bureš *et al.*, 2024), a process that occurs less frequently or is even absent in symmetrics (Barker and Wolf, 2010; Clark *et al.*, 2016; Szövényi *et al.*, 2021).

### Centromere drive preferring larger centromeres may be the master mechanism facilitating post-polyploid diploidization

Centromere drive’s preference for larger centromeres, which can accommodate larger cargo, favours the enlargement of chromosomes through fusions and the proliferation of repetitive DNA. As fusion chromosomes are longer and heavier, they require centromeres of compatible size. Therefore, chromosomes with larger centromeres, which are favoured by centromere drive and can carry a larger cargo, can facilitate the formation and stabilization of fusion chromosomes. In addition, repetitive sequences of larger centromeres/chromosomes provide an additional substrate for ectopic recombination between non-homologous chromosomes, making chromosome fusions even more likely. As deleterious hitchhiking mutations associated with centromere drive are typically located in (peri)centromeric regions (Bureš and Zedek, 2014), chromosome fusions accompanied by the loss of repeat-rich (peri)centromeres (e.g. end-to-end fusions and Robertsonian translocations; Lysak, 2014, 2022; Mandáková and Lysak, 2018) will also mediate the removal of deleterious hitchhikers. Thus, with its ability to remove hitchhikers, PPD could also be perceived as a self-cleaning process of centromere drive.

Centromere drive’s preference for smaller centromeres could hinder PPD-related chromosome fusions and/or facilitate a reduction in chromosome size because smaller centromeres can accommodate a smaller cargo. This reduction could be achieved by removing redundant DNA (mostly repeats), by centric fission of (sub)metacentric chromosomes and, hypothetically, by *de novo* formation of a second centromere followed by fission of the dicentric chromosome.

In contrast, symmetrics lack opportunities for centromere drive, resulting in more stable (less frequently rearranged) chromosomes, genomes and karyotypes that evolve at a slower pace, including the repetitive genome fraction (Wagner and Wagner, 1980; Renzaglia *et al.*, 1995; Nakazato *et al.*, 2008; Clark *et al.*, 2016; Bowman *et al*., 2017; Liu *et al.*, 2019; Elliott *et al.*, 2022; Kinosian *et al.*, 2022; Linde *et al*., 2023). Interestingly, the repeats in plant symmetrics appear to be evenly distributed along chromosomes (Szövényi *et al.*, 2021; Fang *et al.*, 2022; Marchant *et al.*, 2022; Cui *et al.*, 2023; Zhou *et al.*, 2023), without the peaks in the (peri)centromeric regions typical of asymmetrics (Lin *et al.*, 2021; Szövényi *et al.*, 2021; Hou *et al.*, 2022; Santangelo *et al.*, 2023). This suggests that residing close to the centromeres does not increase the chances of repeats being transmitted to the next generation in symmetrics. However, one would still expect an accumulation of fern TEs around centromeres due to lower recombination in those regions, which would prevent their removal. The homogeneous TE distribution along chromosomes in symmetrics thus indicates that the rate of TE removal between centromeres and chromosome arms is not significantly different, a pattern also observed in soybean (Du *et al.*, 2010). As there is no centromere drive for hitchhiking, symmetrics do not require PPD to eliminate negative hitchhiking mutations. Moreover, because they lack centromere drive, non-seed plants do not have a mechanism that would facilitate PPD even if they needed it.

### Conclusions, alternative explanations and future perspectives

The dynamics of centromere size, propelled by centromere drive, would gradually lead to erosion of the positive correlation between genome size and chromosome number over geological time. The centromere drive causing the negative correlation in asymmetrics would be similar to the holokinetic drive proposed for clades with holocentric chromosomes (Bureš and Zedek, 2014), where the entire chromosome acts as a centromere (Melters *et al.*, 2012; Zedek and Bureš, 2018; Mandrioli and Manicardi, 2020), and negative correlations are often present even within genera (Lipnerová *et al.*, 2013; Záveská Drábková, 2013; Bureš and Zedek, 2014; Veleba *et al.*, 2017; Burchardt *et al.*, 2020; Elliott *et al.*, 2022).

It is important to note that centromere drive not only propagates detrimental mutations that can, for instance, reduce seed set (Fishman and Kelly, 2015), but also adversely affects male fertility (Daniel, 2002; Fishman and Saunders, 2008; Finseth *et al.*, 2021). Countermeasures against its deleterious consequences include evolutionary adaptations in kinetochore proteins such as CENH3 that mitigate the impact of centromere drive by ensuring balanced chromosome segregation (Malik and Henikoff, 2003; Talbert and Henikoff, 2022). Alternatively, a reversal of the spindle polarity could potentially flip centromere drive’s preference for larger or smaller centromeres, which is hypothesized to have occurred multiple times during mammalian evolution (Pardo-Manuel de Villena and Sapienza, 2001; Malik and Henikoff, 2003). The switch from a preference for smaller to larger centromeres could be beneficial or even necessary for PPD-driven removal of hitchhikers.

**Fig. 2.**
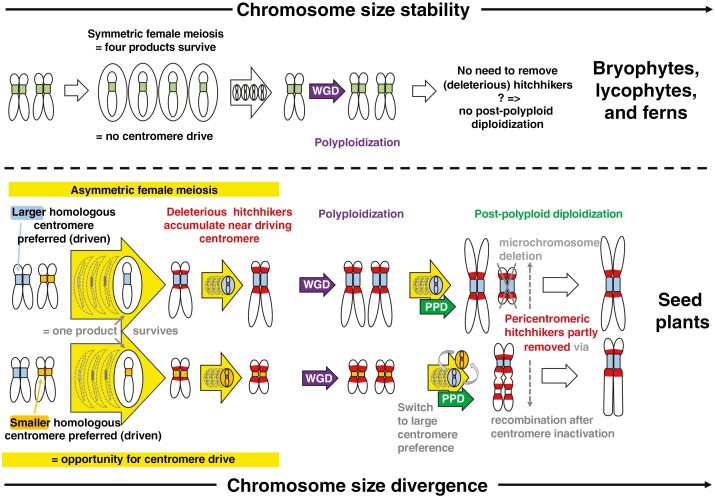
An idealized model of chromosome and genome size evolution in symmetrics and asymmetrics, taking into account the absence or presence of centromere drive (large yellow arrows). It explains the distinct pathways of genome and karyotype evolution in symmetric (bryophytes, lycophytes, ferns) and asymmetric (angiosperms, gymnosperms) lineages, highlighting the role of whole-genome duplication (WGD; purple arrows) and post-polyploid diploidization (PPD; green arrows) in the (in)stability of chromosome number and size. The upper panel shows the situation in bryophytes, lycophytes and ferns (symmetrics). As there is no centromere drive for hitchhiking, symmetrics do not need PPD to eliminate deleterious hitchhikers (in red in the bottom panel). This leads to more stable chromosomes and karyotypes whose evolution is driven primarily by WGDs. The bottom panel shows the situation in angiosperms and gymnosperms (asymmetrics). Centromere drive’s preference for larger (blue) or smaller (orange) centromeres leads to larger or smaller chromosomes, respectively, because the size of centromeres determines the cargo size they can accommodate during cell division. Simultaneously, deleterious hitchhikers (in red) accumulate over time. After a WGD, PPD removes the hitchhikers through Robertsonian translocations or end-to-end chromosome translocations, increasing chromosome size and decreasing chromosome number. In the case of the initial centromere drive’s preference for smaller centromeres, a switch to preferring larger centromeres may be helpful or necessary to remove the hitchhikers.

It should also be noted that any mechanism that can drive alterations in centromere/kinetochore size to fixation, whether natural selection or genetic drift, would affect the evolution of chromosome and genome size. Spores from cryptogamous plants (symmetrics) can be carried over vast distances by wind (Barrington, 1993; Kessler, 2010). Compared to angiosperms and gymnosperms (asymmetrics), this ability potentially enlarges their population sizes and enhances the influence of natural selection over genetic drift. Chromosome (and genome) size growth, e.g. via proliferation of TEs, could thus be under tighter control (Lynch and Conery, 2003) and therefore slower in symmetrics than in asymmetrics, regardless of whether centromere drive is involved. Although we cannot exclude the possibility that the differences in chromosome size evolution between asymmetrics and symmetrics are due to some other mechanisms, our model involving centromere drive is capable of explaining multiple phenomena in a single framework (Fig. 2). An ideal model to study the role of centromere drive in the evolution of chromosome and genome size would be a group of closely related species that differ only in the presence or absence of meiotic asymmetry. Research could focus on several angiosperm species reported to exhibit multisporic (bi-, tetra-) embryo sac development (Schnarf, 1936; Maheshwari, 1937). If the future egg megaspore in these species were selected randomly, they could potentially serve as a symmetric angiosperm model. Future research should also prioritize the investigation of centromeres and chromosomal evolution in clades with symmetric meiosis, such as ferns, bryophytes and lycophytes, which are still significantly understudied in this respect, to fully elucidate the true extent of the importance of centromere drive in the evolution of plant genome size.

## SUPPLEMENTARY INFORMATION

Supplementary data are available online at https://academic.oup.com/aob and consist of the following:

Table S1: List of analysed species with values of traits of interest and their sources. Table S2: List of analysed clades with values of analysed traits. Table S3: CENH3 codon alignments for each clade obtained using the MAFFT algorithm. Table S4: Trees used in the analyses in Newick format. Table S5: N-terminal parts of CENH3 for each clade. Table S6: C-terminal parts of CENH3 for each clade. Table S7: OUwie model parameters. Table S8: Results of phylogenetic generalized linear models (PGLS). Figure S1: Diagram schematically depicting the inference of the rates of chromosome size evolution. Figure S2: Box plots showing differences between asymmetrics and symmetrics in the branch-level from aBSREL (a, c, e) and codon-level from MEME (b, d, f) dN/dS ratios of full-length, N-terminal and C-terminal parts of CENH3. Figure S3: Bootstrapped values of the frequency of positive selection acting on the C-terminal part of CENH3 in clades with female asymmetric meiosis (θ_A_ = 0.0029) and symmetric meiosis regardless the sex (θ_S_ = 0.0001) under the OUMV model. File S1: CENH3-gene trees with paralogous groups

mcae149_Suppl_Supplementary_Tables_S1-S8

mcae149_Suppl_Supplementary_File_S1

mcae149_Suppl_Supplementary_Figure_S1

mcae149_Suppl_Supplementary_Figure_S2

mcae149_Suppl_Supplementary_Figure_S3
