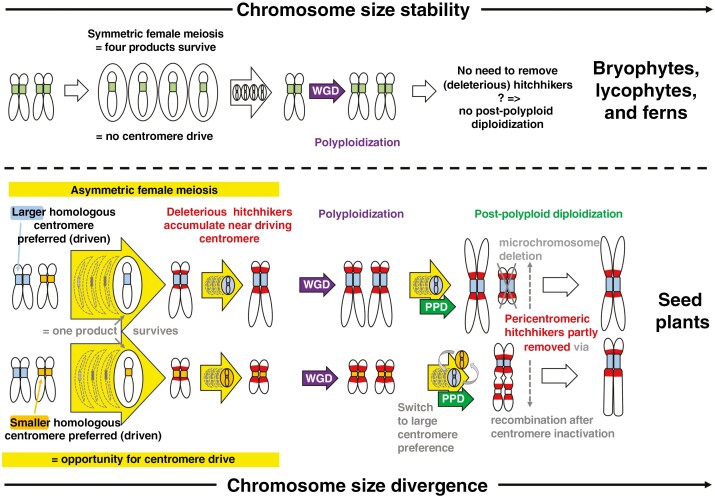# Correction to: Centromere drive may propel the evolution of chromosome and genome size in plants

**DOI:** 10.1093/aob/mcae200

**Published:** 2024-11-16

This is a correction to: Klára Plačková, Petr Bureš, Martin A Lysak, František Zedek, Centromere drive may propel the evolution of chromosome and genome size in plants, *Annals of Botany*, 2024;, mcae149, https://doi.org/10.1093/aob/mcae149

The original Figure 2 contained an error in the representation of the microchromosome deletion. The revised Figure 2 now accurately depicts the microchromosome deletion during the process of post-polyploid diploidization with the intended level of detail.