# Supplementary material for: Centromere drive may propel the evolution of chromosome and genome size in plants
Source: Ann Bot. 2024 Aug 28;134(6):1067–76. doi: 10.1093/aob/mcae149 (PMC11687628; doi:10.1093/aob/mcae149)

Centromere drive may propel the evolution of chromosome and genome size in plants

Klára Plačková, Petr Bureš, Martin A. Lysak, František Zedek

Supplementary File S1 – CENH3-gene trees with paralogous groups

Amaranthaceae


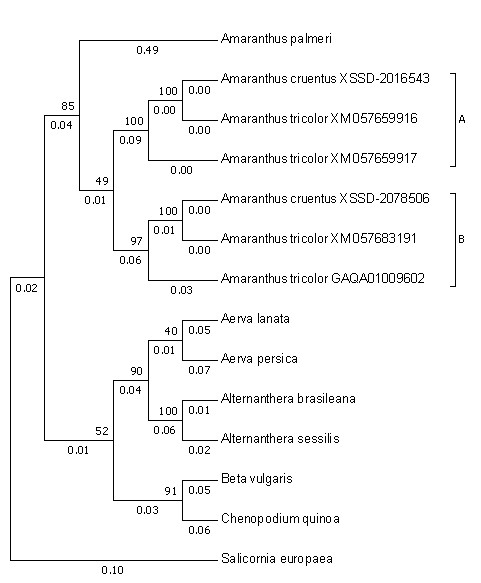


Anthocerotophyta


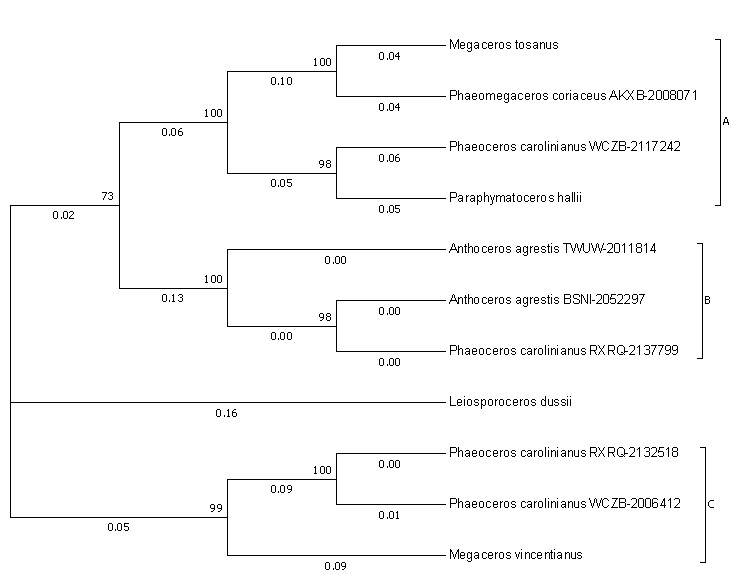


Asteraceae


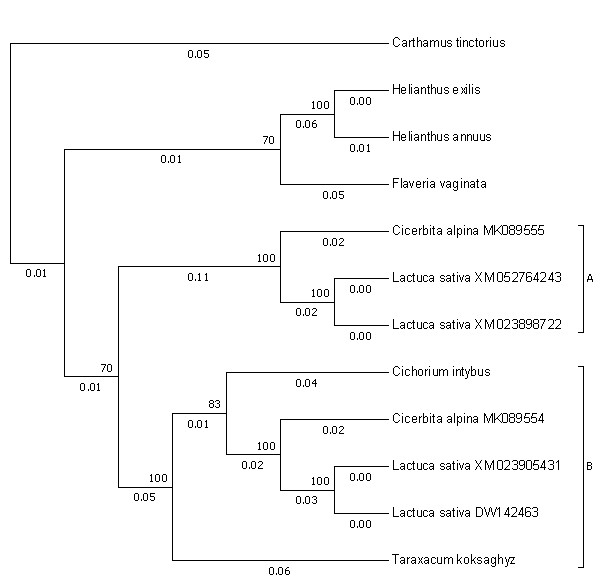


Brassicaceae


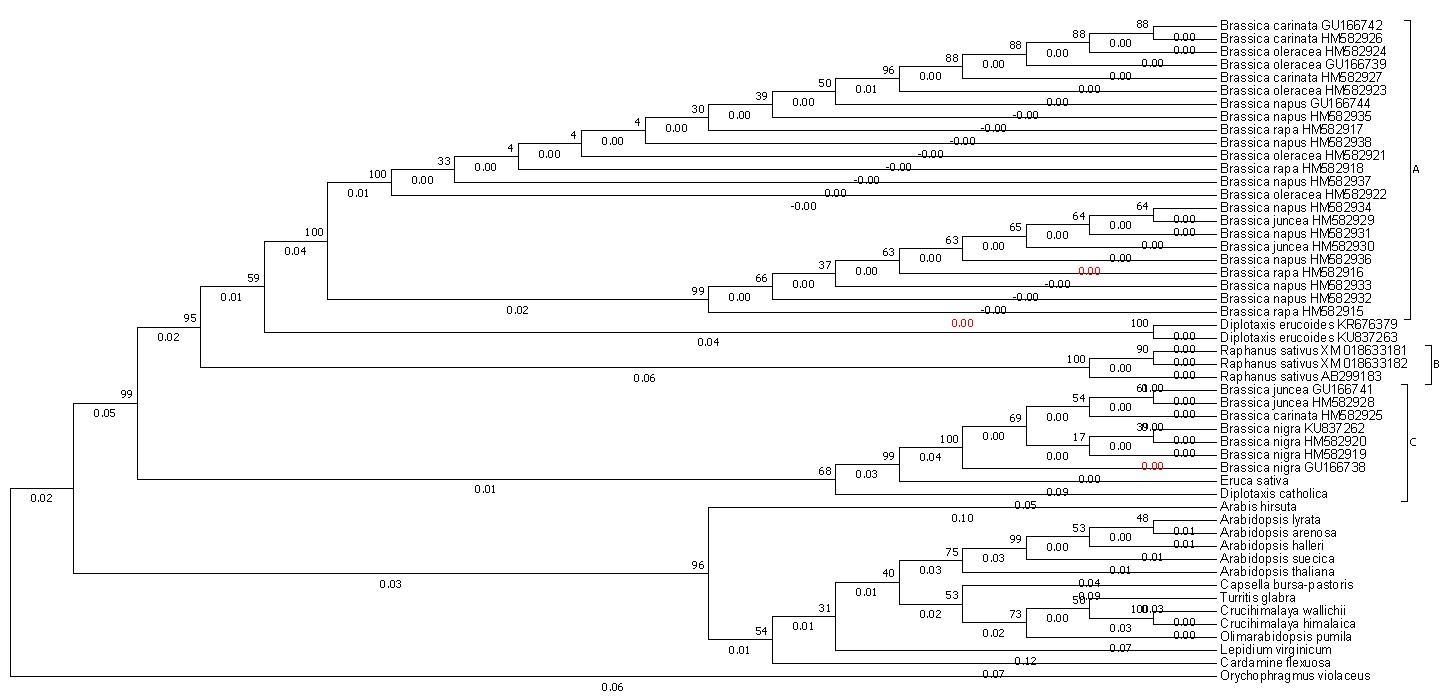


Convolvulaceae


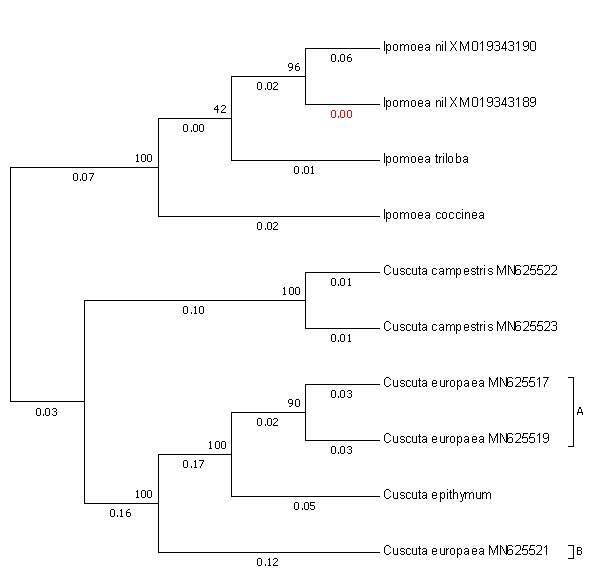


Cucurbitales


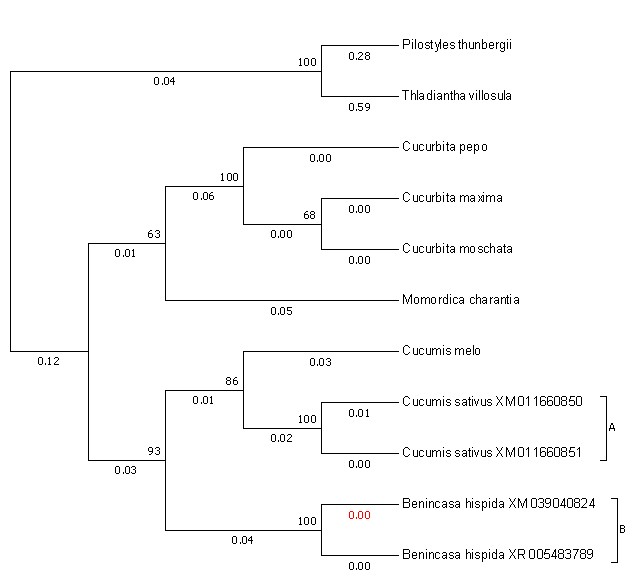


Cupressaceae


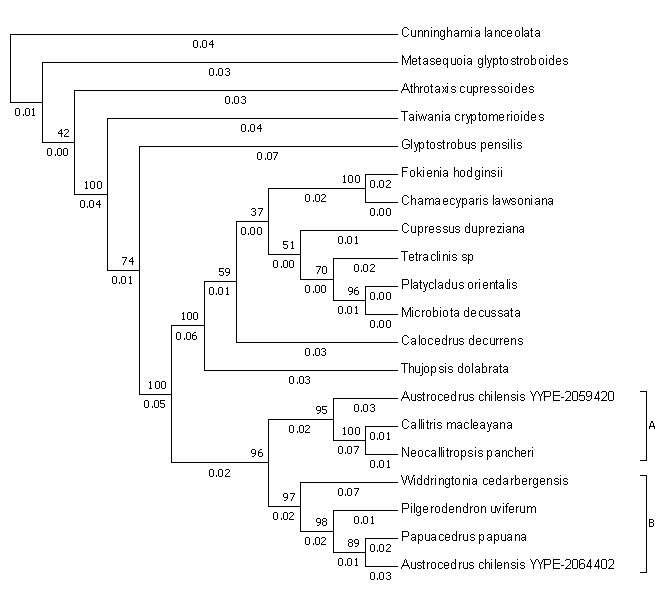


Cyperaceae


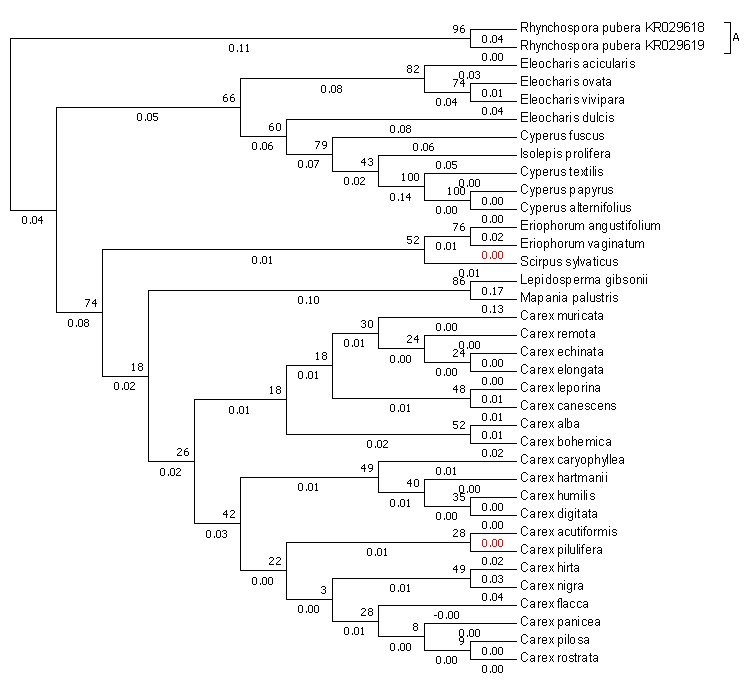


Fabaceae


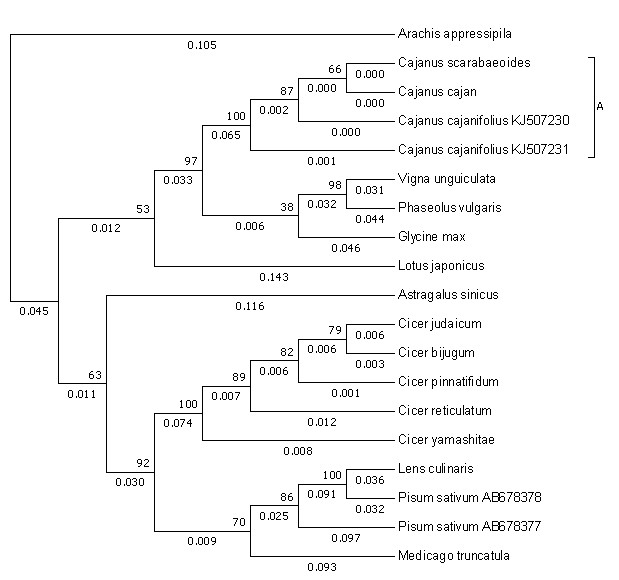


Fagales


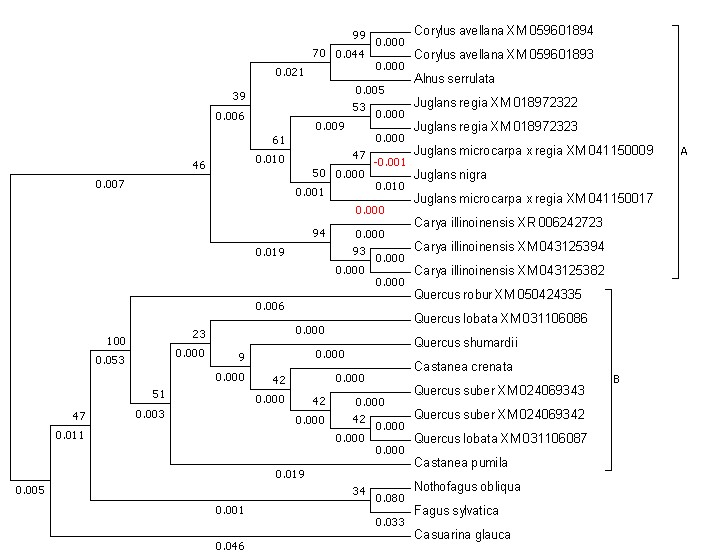


*Luzula*


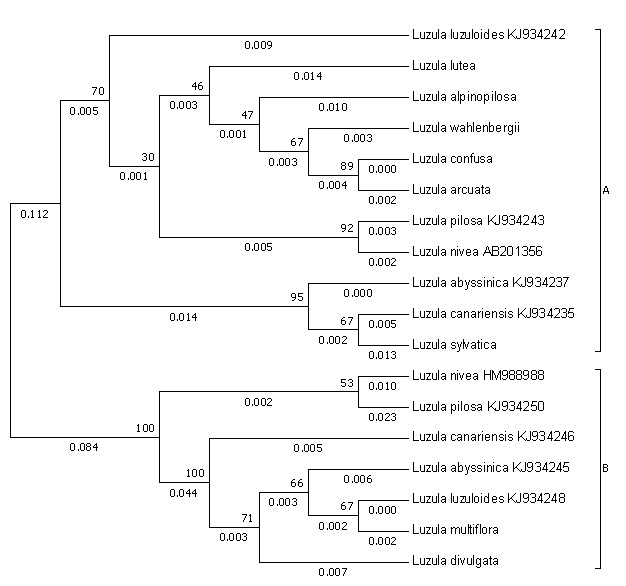


Malvaceae


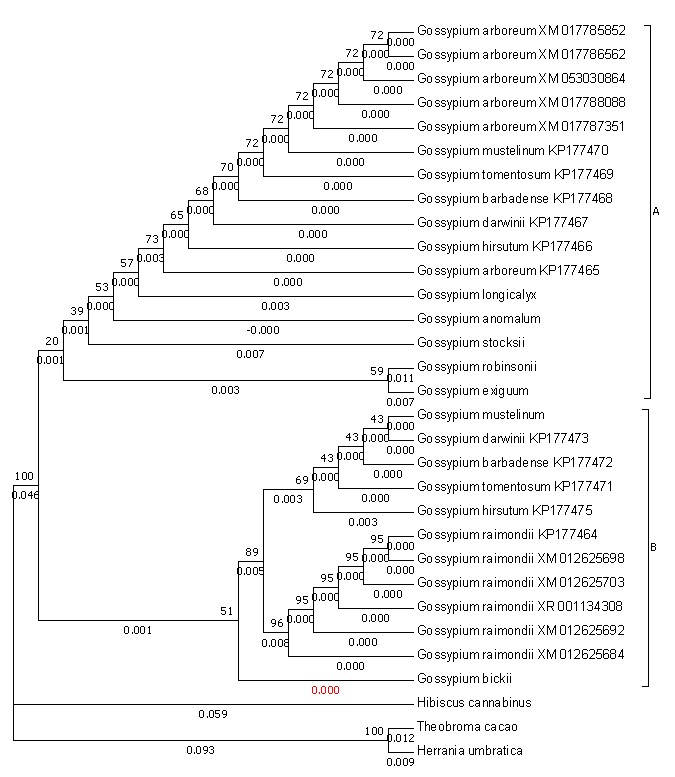


Pinaceae


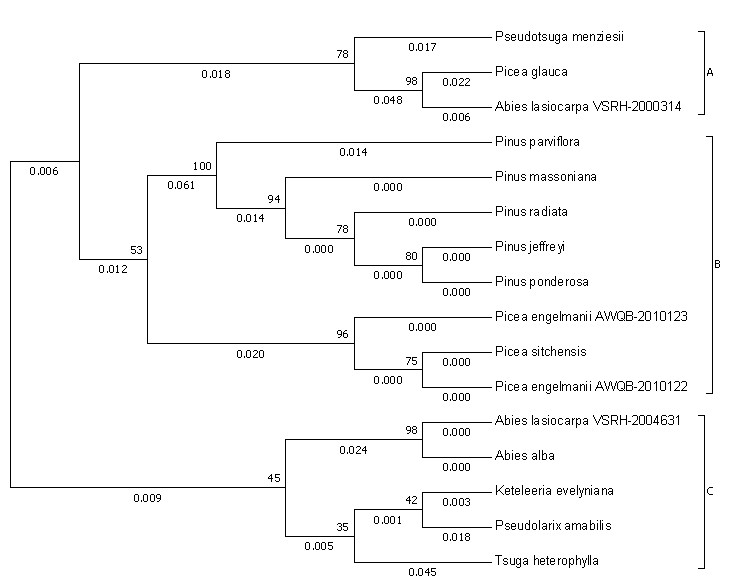


Poaceae


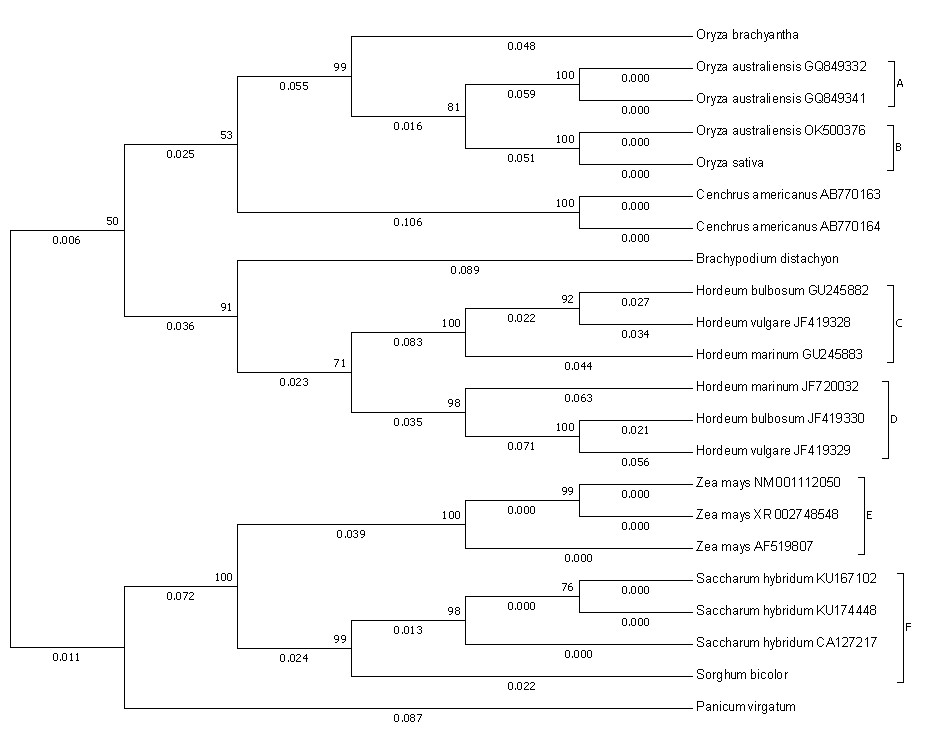


Rosaceae


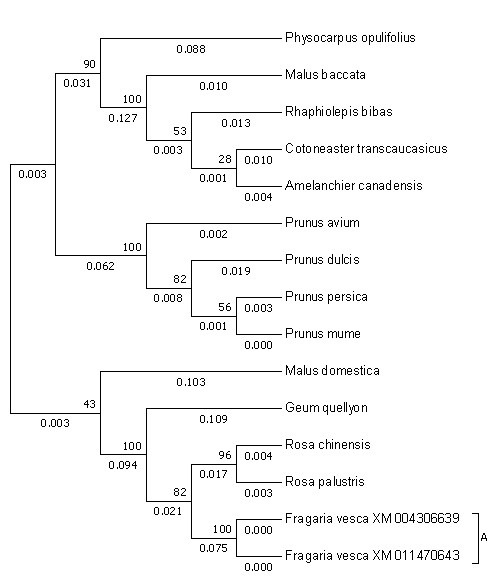


Solanaceae


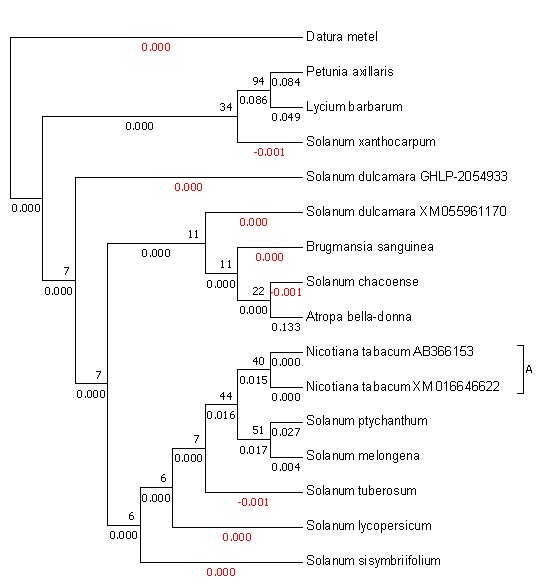

Supplement: mcae149_Suppl_Supplementary_File_S1 [file mcae149_suppl_supplementary_file_s1.docx]
