# Supplementary figures and images for: Centromere drive may propel the evolution of chromosome and genome size in plants
Source: Ann Bot. 2024 Aug 28;134(6):1067–76. doi: 10.1093/aob/mcae149 (PMC11687628; doi:10.1093/aob/mcae149)

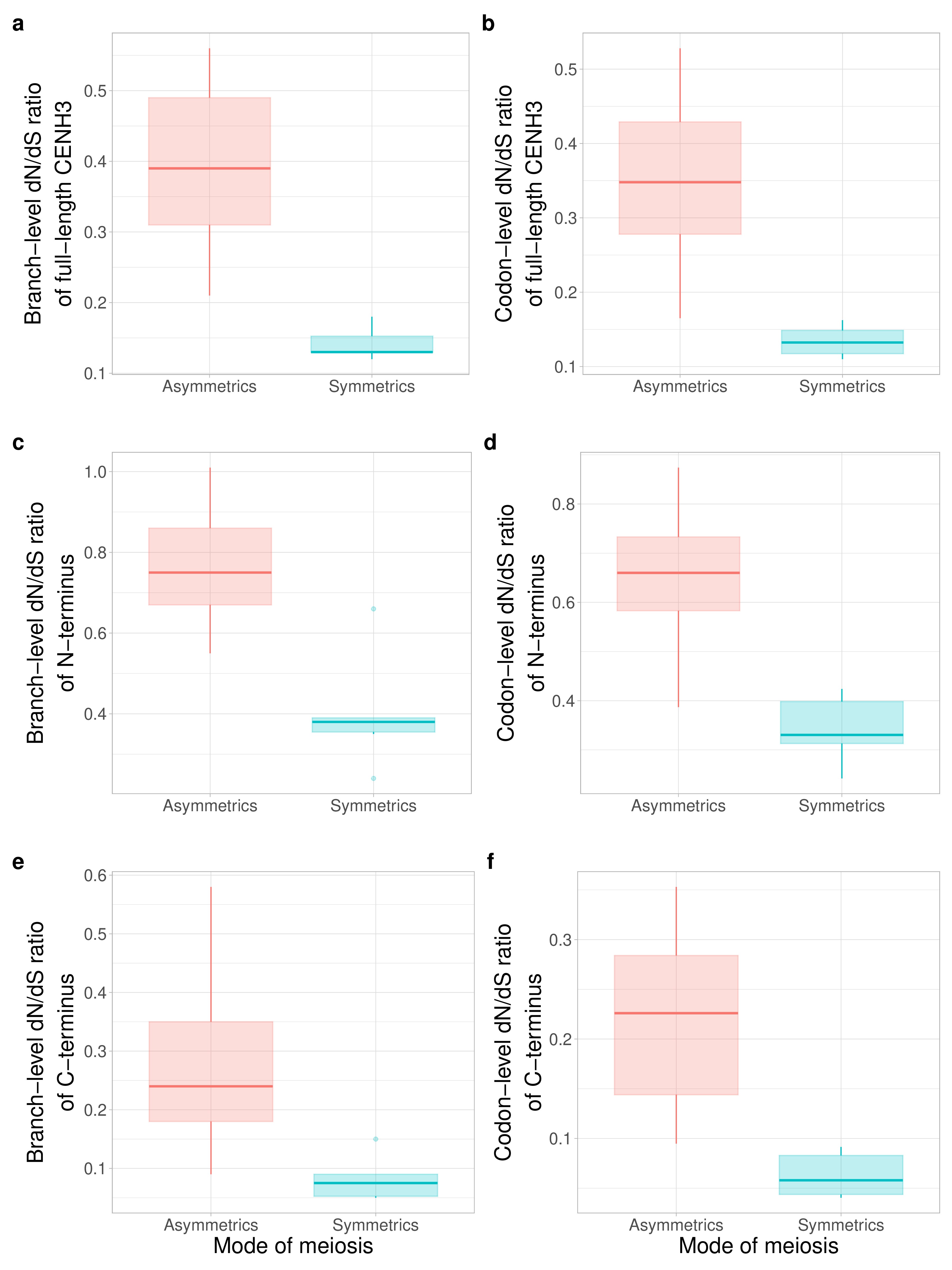

Supplement: mcae149_Suppl_Supplementary_Figure_S1 [file mcae149_suppl_supplementary_figure_s1.jpeg]

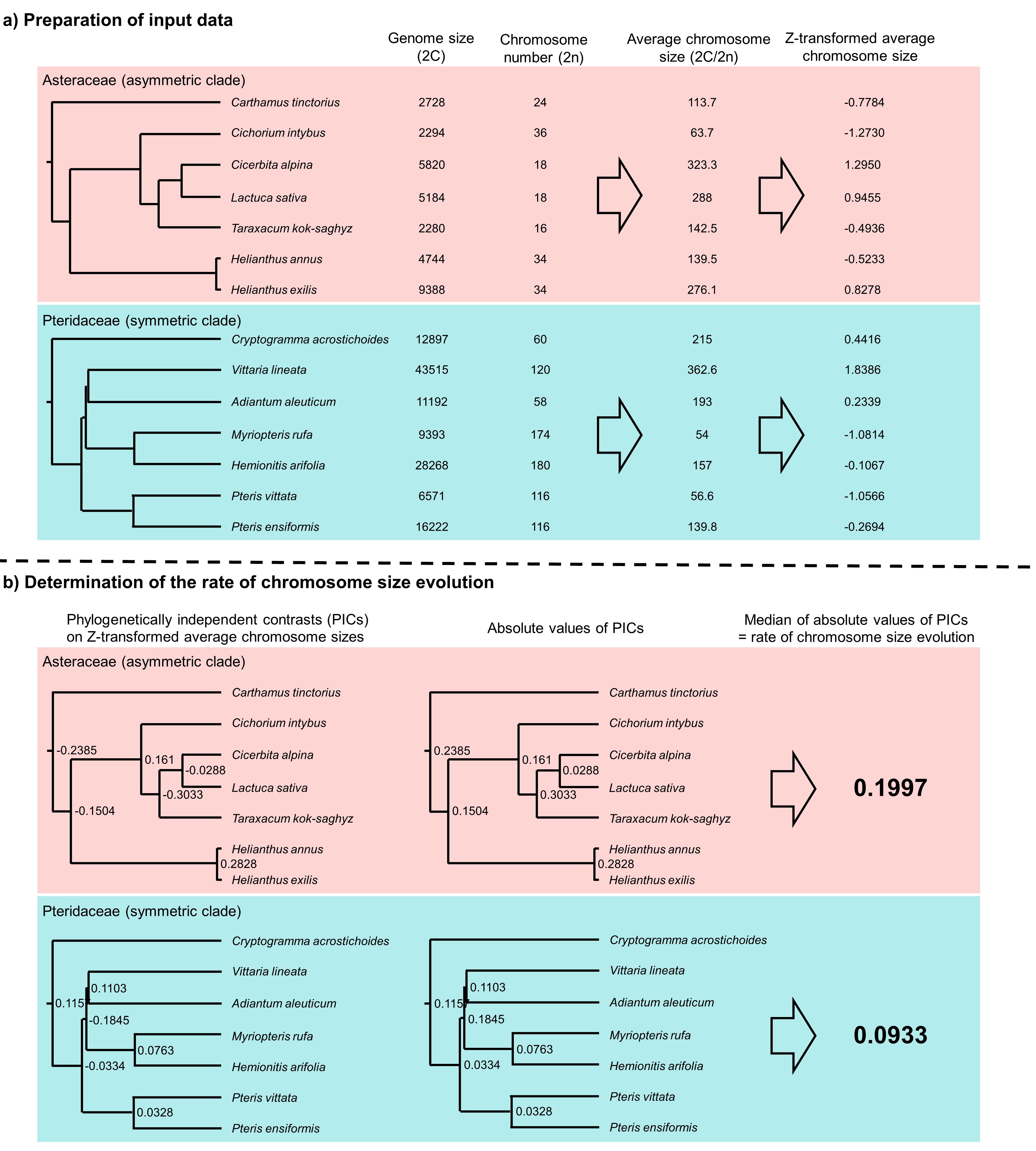

Supplement: mcae149_Suppl_Supplementary_Figure_S2 [file mcae149_suppl_supplementary_figure_s2.jpeg]

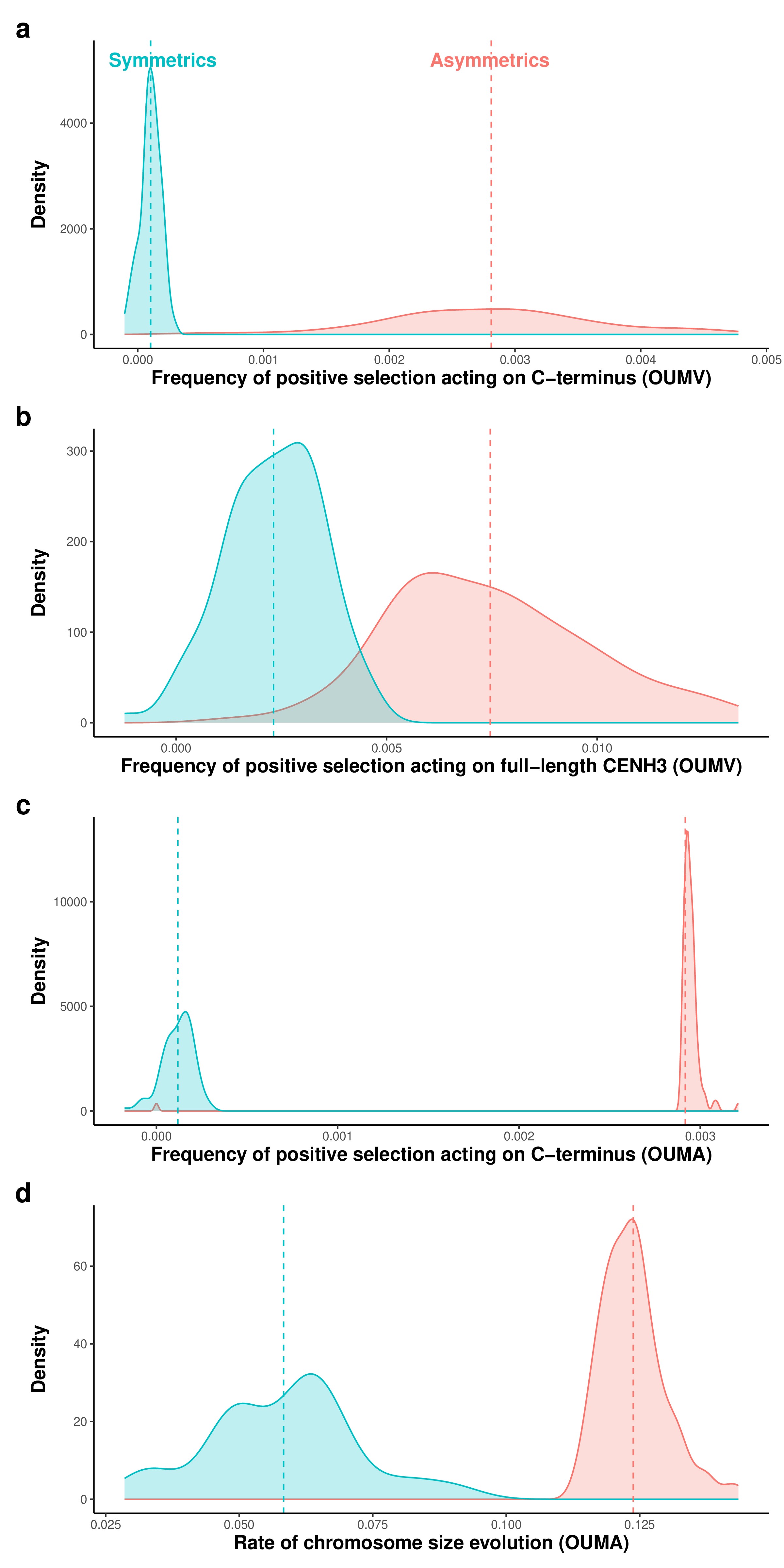

Supplement: mcae149_Suppl_Supplementary_Figure_S3 [file mcae149_suppl_supplementary_figure_s3.jpeg]
